# Cold stress impairs CurT accumulation and thylakoid architecture in *Synechocystis* sp. PCC 6803

**DOI:** 10.3389/fpls.2026.1876046

**Published:** 2026-06-26

**Authors:** Victoria Julia Christine Holzer, Anne-Christin Pohland, Jörg Nickelsen, Marcel Dann, Matthias Ostermeier

**Affiliations:** 1 Plant Molecular Biology, Faculty of Biology, Ludwig-Maximilians University Munich, Munich, Germany; 2 Bio-Inspired Energy Conversion, Technical University of Darmstadt, Darmstadt, Germany

**Keywords:** cold stress, CurT, cyanobacteria, environment, *Synechocystis*, thylakoids

## Abstract

Low-temperature stress affects the thylakoid membrane system in both cyanobacteria and chloroplasts. In this study, a previously unrecognized link between cold-stress acclimation and the thylakoid-shaping CurT abundance in *Synechocystis* sp. PCC 6803 is identified. The results demonstrate that thylakoid architecture and photosynthetic performance are highly sensitive to low-temperature conditions. Furthermore, cold stress appears to impose a common physiological bottleneck that masks CurT-specific differences. Together, our findings support a model in which downregulation of CurT contributes to membrane robustness when thylakoid fluidity and curvature are challenged by low temperature. This study broadens the current understanding of cyanobacterial cold acclimation and identifies CurT as a factor linking membrane architecture to stress adaptation.

## Introduction

Oxygenic photosynthesis has shaped global biogeochemical cycles and all life on Earth (Fournier et al., 2021). Environmental stress however affects both the molecular components of the photosynthetic machinery and the intracellular thylakoid membrane (TM) system that hosts key protein complexes of the photosynthetic electron transport chain in cyanobacteria and chloroplasts (Rippka et al., 1974; Liu, 2016). In particular, low temperatures affect TMs and their functional components, as it imposes constraints on membrane fluidity and the structural organization of photosynthetic complexes within the TM (Heydarizadeh et al., 2013; Mironov and Los, 2016). Such constraints can only be partially alleviated through modulation of membrane lipid fatty acid saturation state and corresponding increases in TM fluidity (Los et al., 2013; Rachedi et al., 2020), as protein *de-novo* biosynthesis and pre-protein processing, especially for repair of damaged photosystem II (PSII), is strongly impaired at low temperatures (Kanervo et al., 1997; Allakhverdiev and Murata, 2004).

Correspondingly, cyanobacteria as well as chloroplasts have evolved various cellular strategies to cope with cold stress. For instance in chloroplasts cold stress leads to starch depletion, chloroplast swelling, reduction of thylakoid grana stacks, accompanied with distorted and less tightly appressed TMs with enlarged thylakoid lumen (Martínez-Peñalver et al., 2012). These ultrastructural changes are suggested to aid in lowering photosynthetic electron output, thus protecting plants from oxidative stress and the negative effects of lipid peroxidation in the membranes (Venzhik et al., 2019). Meanwhile, in cyanobacteria, cold stress has been reported to primarily alter the expression and structural integrity of pigment-protein complexes such as photosystems and phycobilisomes, which in turn affects the accumulation of chlorophyll *a* and phycobiliproteins in *Spirulina* and marine *Synechococcus* species (Kumar et al., 2011; Mackey et al., 2013). Reduced temperatures further modulate carotenoid biosynthesis and stability (Zakar et al., 2017), all of which likely affect photosynthetic light harvesting and energy conversion. Whether chloroplast-related rearrangements of the TM ultrastructure occur in cyanobacteria as well, is unclear to date. In the mesophilic model cyanobacterium *Synechocystis* sp. PCC 6803, which grows productively at a broad temperature range of 25-40 °C (Inoue et al., 2001), no pronounced effect on cell morphology or the structure of membrane systems was observed following a temperature drop from 34 °C (moderate heat) to 22 °C (moderate cold) (Keta et al., 2025). Still, for severe cold stress, no such study has been conducted yet.

In *Synechocystis*, the transmembrane protein CurT (ORF *slr0483*) has been identified as a key factor of TM ultrastructure (Armbruster et al., 2013; Heinz et al., 2016). In chloroplasts, loss of the CurT-homologue CURT1 leads to the loss of TM grana stacking, while in *Synechocystis* loss of CurT results in irregular thylakoid arrangement and complete absence of TM curvature towards the plasma membrane (PM) in thylakoid convergence zones (TCZs) (Armbruster et al., 2013; Heinz et al., 2016; Pribil et al., 2018) which have been linked to PSII assembly and repair (Rengstl et al., 2011; Heinz et al., 2016; Ostermeier et al., 2025).

Here, we investigated the role of CurT under enhanced cold stress (15 °C) in wild type (WT) vs. CurT knockout and overexpression lines (Dann et al., 2025; Ostermeier et al., 2026). The data reveals that 15 °C cold stress induces a down-regulation of cellular CurT protein levels in WT compared to control conditions, accompanied by a strong rearrangement of membrane architecture. Moreover, phenotypes of WT and CurT expression-level mutants (*i.e.*, knockout and overexpression) converge to each other with regard to growth, photosynthetic protein abundance, PSII performance, and ultrastructural membrane organization in the cold. These findings for the first time show chloroplast-like ultrastructural acclimation responses of the cyanobacterial TM system to severe cold, and point towards a novel functional connection between cellular CurT levels, stabilization of membrane architecture, and photosynthetic performance at low temperature conditions.

## Methods

### Cyanobacterial strains and culture conditions

Experiments were conducted with glucose-tolerant *Synechocystis* WT and CurT expression strains established in Dann et al. (2025). For KO the CurT coding sequence (ORF *slr0483*) has been replaced with a SpecR resistance cassette, for OE the entire CurT gene has been inserted into a genomic neutral site (ORF *slr0168*) by homologous recombination. Liquid cultures were grown at 25 °C under continuous illumination with 50 μmol photons m^-2^ s^-1^ white fluorescent light (shaker cultures: 5000 K, FHF32EX-N-HX-S; plate cultures: 4000 K; MonotaRO Co. Ltd., Hyogo, Japan) at 65 rotations per minute orbital shaking in BG11 photoautotrophic medium (Rippka et al., 1979). To obtain temperature-dependent growth differences, WT and *CurT* expression strains were inoculated at OD_720_ = 0.05 and grown at 25 °C or 15 °C in a Multi-Cultivator MC 1000-OD (Photon Systems Instruments, Drásov, Czech Republic) under 50 µmol photons m^-2^ s^-1^ of warm-white LED light and atmospheric aeration with apparent OD_720_ being recorded in 60-min intervals using the built-in MC 1000-OD photometer.

Because optical density measurements are influenced by cell size and scattering properties, OD_720_ values are reported as apparent culture density and were primarily used to compare growth dynamics between strains within a given temperature regime.

### Cyanobacterial protein extraction, immunodetection, and quantification

For immunodetection, precultures were grown at 25 °C, in BG11 photoautotrophic medium, 40 μmol photons m^-2^ s^-1^ and continuous shaking (5000 K, FHF32EX-N-HX-S; plate cultures: 4000 K; MonotaRO Co. Ltd., Hyogo, Japan). After 3 days, cultures were split to 25 °C or 15 °C with an initial OD_720_ = 0.05 and grown to mid-exponential growth. Synechocystis whole-cell protein extracts were prepared as previously described (Ostermeier et al., 2022). 30 μg of total protein extract were separated by 15% SDS PAGE Tris-Glycine gels and blotted onto a Nitrocellulose membrane (pore size 0.45 μm) at 100 V for 60 min (Tank blot). The CurT protein was immuno-detected using primary antibody serum raised against *Synechocystis* CurT (Heinz et al., 2016) (LMU Munich) at a 1:1000 dilution. D1, PratA and S1 were immuno-detected using polyclonal primary antibody (Agrisera, Sweden). Immunoblot ECL signals were detected using horse-radish-peroxidase conjugated Goat anti-Rabbit IgG AS09 602 (Agrisera, Sweden) as secondary antibody, quantified using ImageJ (Schneider et al., 2012), and normalized to the intensity of the loading control (S1).

### Whole cell absorbance spectra

For whole-cell absorbance spectra, a cell suspension with an OD720 of 3 was prepared in a final volume of 100 or 200 µL. The suspension was mixed with an equal volume of PBST (1× PBS buffer containing 0.1% Tween 20) and centrifuged at 3, 500 × g at room temperature. The resulting pellet was resuspended in 100 or 200 µL of PBST buffer. The absorption spectrum was measured between 350 nm and 800 nm using microvolume mode (FastGene^®^ Photometer NanoSpec (FG-NP01; NIPPON Genetics Europe GmbH, Düren, Germany). The offset value at 800 nm was subtracted from all data points in the spectrum, and the curves were normalised at an OD of 680 nm.

### Fluorescence analysis to estimate the maximum and effective quantum yields

To assess relative changes in chlorophyll fluorescence characteristics of *Synechocystis* cells, fluorescence light and OJIP [fluorescence light and OJIP curves (https://www.cyano.tools/OJIP_data_analysis); Strasser and Govindjee, 1992] curves were obtained using an AquaPen (AquaPen AP110-C, Photon Systems Instruments, Drásov, Czech Republic). Measurements were performed using the manufacturer’s [630 nm] excitation source. Because fluorescence signals in cyanobacteria contain contributions from PSII as well as PSI and, depending on excitation wavelength, phycobilisomes, the resulting fluorescence parameters are interpreted as apparent rather than absolute measures of PSII photochemical performance. The measuring pulses were set at 10%, while the saturating light pulses were set to 50%. A 2 mL sample of *Synechocystis* culture, adjusted to an OD_720_ of 0.4 was measured. Prior to the measurement, cultures were dark-acclimated for 10 minutes. To determine the maximum and effective quantum yield under varying light intensities, a predefined light curve three (LC3; light intensities in μmol photons m^-2^ s^-1^: 10, 20, 50, 100, 300, 500, 1000). In addition, Chl *a* fluorescence transients were analysed to assess PSII activity and the redox state of the plastoquinone (PQ) pool.

### Transmission electron microscopy

For ultrastructural analysis of *Synechocystis* cells, sample preparation was performed as described in Lübben et al. (2024). In brief, cryofixation performed with EM HPM100 (Leica Microsystems, Wetzlar, Germany) was followed by freeze-substitution in A.O.U.H. solution at –90 °C (acetone containing 0.2% [w/v] OsO_4_, 0.1% [w/v] uranyl acetate, and 9% [v/v] H_2_O) for 42 hours. After embedding of samples in Epon 812 for 16 hours at 63 °C, ultrathin sectioning of 70 nm (ultra 35°, 3.0 mm, DiATOME) was conducted via an Ultracut E ultramicrotome (Leica Microsystems). Ultrathin sections were post-stained with lead citrate and visualized with a Zeiss EM 912 transmission electron microscope (Zeiss, Oberkochen, Germany) equipped with an integrated OMEGA energy filter and CCD camera (Tröndle Restlichtverstärkersystem, Moorenweis, Germany).

### Statistical analysis

All quantitative data are presented as means ± standard deviation of independent biological replicates, as indicated in the respective figure legends. Differences between groups were evaluated using two-sided unpaired Student’s t-tests. Exact sample sizes (n) are reported in the corresponding figure legends. Statistical significance was accepted at *p* < 0.05.

## Results

### Cold stress affects cellular CurT levels and mitigates growth differences between cells with altered CurT abundance

**Figure 1:**
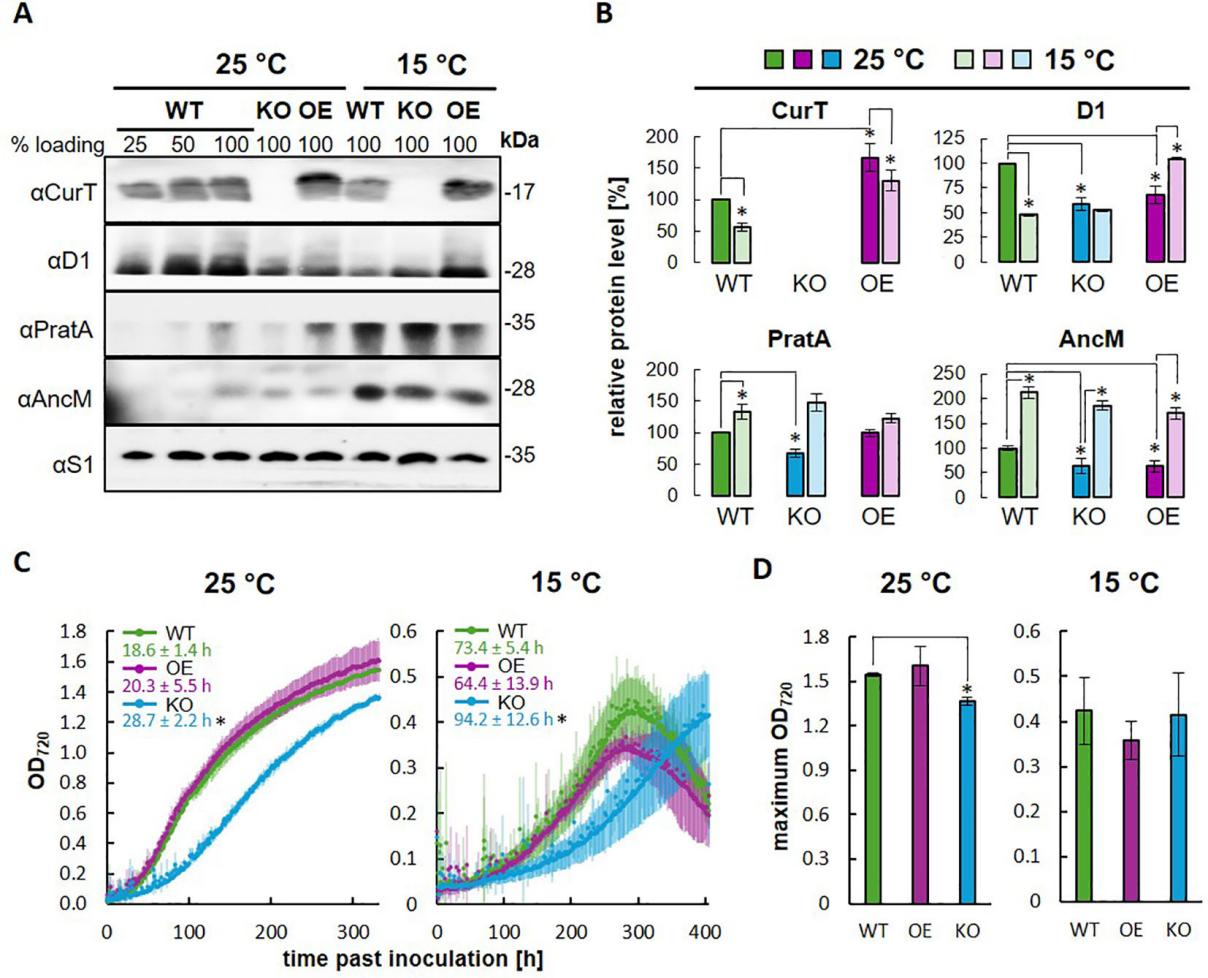
Cold stress affects protein accumulation and attenuates growth differences among strains with varying CurT levels. **(A)** Immunoblot analysis of whole-cell protein extracts with antibodies raised against CurT, AncM, PratA, D1 (PsbA) and S1 (RpsA). Ribosomal protein S1 served as a loading control. **(B)** Selected protein levels at 25 °C and 15 °C relative to 25 °C WT signal at 30 µg total protein (100% loading). Columns represent averages, error bars represent standard deviations of *n* = 3 biological replicates, except for AncM detection (*n* = 2). **(C)** Growth curves of *CurT* expression level mutants at 25 °C (control) and 15 °C (cold stress) at 50 µmol photons m^−2^ s^−1^, monitored as apparent OD_720_ (Multicultivator). Dots represent averages, error bars represent standard deviations of *n* = 4 biological replicates. Average duplication times ± standard deviations are indicated. **(D)** Maximum apparent OD_720_ detected in Multicultivator cultures. As cell size differs between temperature conditions (**Supplementary Figure 1**), OD720 values represent apparent culture density and may not directly reflect cell number. For 15 °C, maxima are derived as averages of 6 data points (*i.e*., 6 h sliding windows) to minimize the effect of detection noise near the culture density maximum. Dots represent averages; error bars represent standard deviations of *n* = 4 biological replicates. WT, wildtype; KO, *CurT* knock-out; OE, *CurT* overexpression. Asterisks indicate statistically significant differences according to two-sided unpaired *t*-test.

*Synechocystis CurT* mutants without (knock-out, KO), or with elevated (overexpression, OE) CurT levels were cultivated in BG11 growth media under photoautotrophic conditions at 25 °C (control) and 15 °C (cold stress). At 25 °C, CurT protein levels reflected the expected genotype-specific differences, with no detectable CurT signal in KO and a strong increase in OE mutant strains (+67%) relative to WT. These differences were accompanied by reduced levels of PSII core protein D1 in both KO (-41%) and OE (-38%) lines as compared to WT. Similarly, abundance of the TCZ-marker protein PratA (Klinkert et al., 2004; Rengstl et al., 2011) was decreased in the KO (-32%) line, whereas OE cells maintained WT-like levels. Levels of the TCZ-PM anchor protein AncM, were reduced in the KO (-36%) line, consistent with previous reports (Heinz et al., 2016; Dann et al., 2025; Ostermeier et al., 2026). However, in contrast to PratA, AncM also declined in the OE (-37%) line (**Figures 1A, B**) relative to WT samples.

Intriguingly, exposure to 15 °C significantly reduces CurT levels in WT cells (-42%) compared to the 25 °C control. In the OE strain, CurT abundance was also significantly reduced upon temperature shift from 25 °C to 15 °C (−22%, ), indicating that cold stress decreases CurT accumulation irrespective of genetic background (**Figures 1A, B**). In the cold, D1 levels sharply decreased in WT (-52%) whereas they remained constant at low level in the KO strain. CurT overexpression however resulted in significantly increased D1 accumulation, restoring D1 levels to values comparable to WT cells grown under control conditions. Thus, unlike several other physiological parameters that converged under cold stress, D1 accumulation remained responsive to CurT abundance. In contrast, both PratA and AncM accumulation increased substantially in all strains under cold conditions (**Figures 1A, B**). Overall, cold stress was found to reduce CurT levels and uncouple D1 accumulation from CurT abundance, while strongly increasing PratA and AncM levels across all strains.

To assess the effects of cold-dependent changes in protein accumulation on a phenotypic level, photoautotrophic growth dynamics and maximum apparent culture densities (OD_720_) were analyzed in the cold. Because cell size affects light scattering, OD_720_-derived parameters should be interpreted as apparent culture density rather than direct measures of cell number. Under photoautotrophic conditions at 25 °C, the CurT-depleted KO mutant showed a growth defect with decreased final culture OD_720_ (-12%) and increased duplication time (+54%) (**Figures 1C, D**), corroborating previous observations in mixotrophic cultures (Heinz et al., 2016; Dann et al., 2025). Meanwhile, under control conditions, the OE strain showed growth similar to WT, with a slightly increased final culture density (**Figures 1C, D**), in line with previous observations (Dann et al., 2025; Ostermeier et al., 2026). Conversely, in the cold, duplication rates of WT and *CurT* KO mutants converged towards each other, and the OE strain showed a slightly lower duplication time as compared to WT (**Figures 1C, D**). Interestingly, OE mutants reached a lower maximum cell culture density than either WT or KO at the 15 °C regime, while the KO strain reached WT-like maximum culture density (**Figures 1C, D**). In sum, this data indicates a reduction of CurT-related differences for both TCZ marker protein accumulation and growth behaviour upon exposure to cold stress.

### Low temperature attenuates CurT-dependent effects on photosynthetic performance

**Figure 2:**
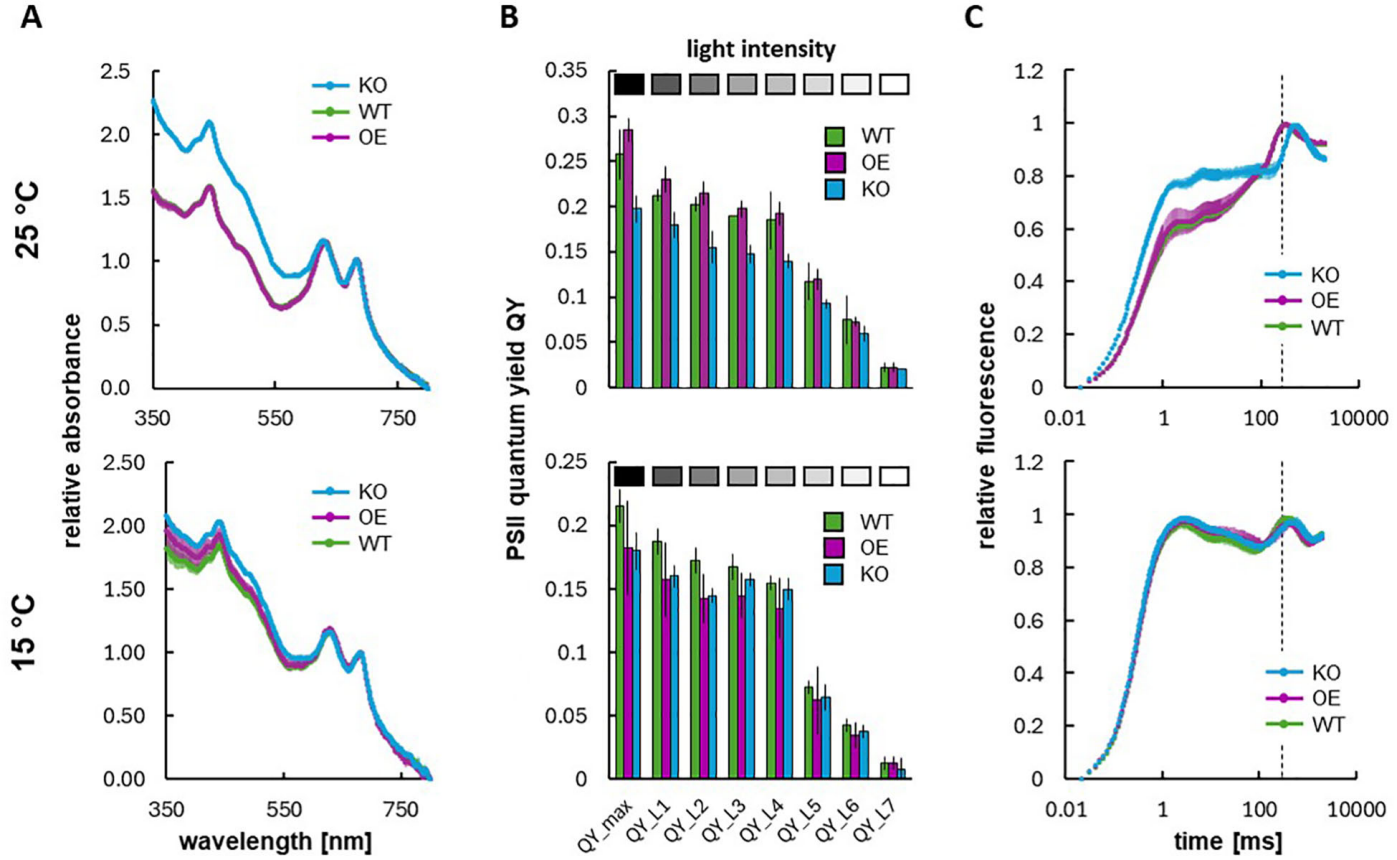
Pigment composition and PSII activity at 15 °C and 25 °C. **(A)** Whole-cell absorbance spectra of cultures grown at 25 °C (top) and 15 °C (bottom). Traces represent averages, shaded areas represent standard deviations of *n* = 4 biological replicates. Spectra are offset at 800 nm and normalized to OD_680_. **(B)** Light-curve assessment of apparent Fv/Fm values (after dark acclimation) and apparent effective fluorescence yields. Owing to contributions of PSI and potentially phycobilisomes to cyanobacterial fluorescence signals, these parameters should not be interpreted as absolute measures of intrinsic PSII quantum yield. Cultures are grown and measured at 25 °C (top) and 15 °C (bottom). Step-wise increase of light intensities from 0 to 1000 µmol photons m^-2^ s^-1^ are indicated. Columns represent averages, error bars represent standard deviations of *n* = 4 biological replicates. **(C)** Chlorophyll fluorescence induction kinetics (OJIP transients) of cultures grown and measured at 25 °C (top) and 15 °C (bottom). Traces represent averages, shaded areas represent standard deviations of *n* = 4 biological replicates. Dashed line indicates timing of control (25 °C) WT P-peak.

To further investigate the physiological effects of cold stress on WT and CurT mutants, culture pigmentation and PSII-related fluorescence parameters were analysed. At 25 °C, the KO mutant exhibits increased apparent whole-cell absorbance in the 350–550 nm region, together with altered photochemistry (**Figures 2A–C**). Notably, these clear genotype-dependent differences diminish in the cold. Instead, whole-cell absorbance spectra of WT, KO, and OE converged, primarily due to increased absorbance in the 350–550 nm range in WT and OE, resembling the KO-phenotype at 15 °C (**Figure 2A**). Fluorescence light curves (**Figure 2B**) were recorded to determine the apparent maximum fluorescence parameter (apparent Fv/Fm) after dark acclimation and corresponding apparent effective fluorescence yields under various actinic light intensities. Under control conditions, the OE strain exhibited apparent Fv/Fm values comparable to that of the WT, with a tendency towards slightly higher values, whereas the KO mutant showed a reduced quantum yield, consistent with previous reports (Ostermeier et al., 2026). With increasing light intensity, the effective quantum yields of all three strains converged, as reported previously (Ostermeier et al., 2026). The lower maximum and effective quantum yields observed in the KO mutant are consistent with impaired PSII electron transfer efficiency (Heinz et al., 2016; Ostermeier et al., 2026). Under cold stress conditions, these differences were no longer observed. Thus, all strains exhibited similarly reduced apparent fluorescence yields, indicating that cold stress strongly affects PSII performance and particularly diminishes the advantage observed in the OE strain under control conditions (**Figure 2B**). To further investigate electron transport and the redox state of the PQ pool, chlorophyll fluorescence induction kinetics (OJIP transients) were measured (**Figure 2C**). Under control conditions, the OJIP curves differed markedly between WT and OE compared to the KO strain. Notably, the J-step (≈2 ms) was higher in the KO strain and the P peak was delayed.

These differences between WT, OE, and the KO strain disappeared under cold stress, with WT and OE exhibiting OJIP characteristics similar to those of the KO mutant under control conditions.

Together, the observed changes in whole-cell absorbance spectra are consistent with alterations in cellular pigmentation and/or light scattering properties under cold conditions in *Synechocystis*. Cold stress further masks CurT-dependent modulation of PSII electron transport kinetics.

### Cold stress leads to disorganized thylakoid architecture and increased cell size

**Figure 3:**
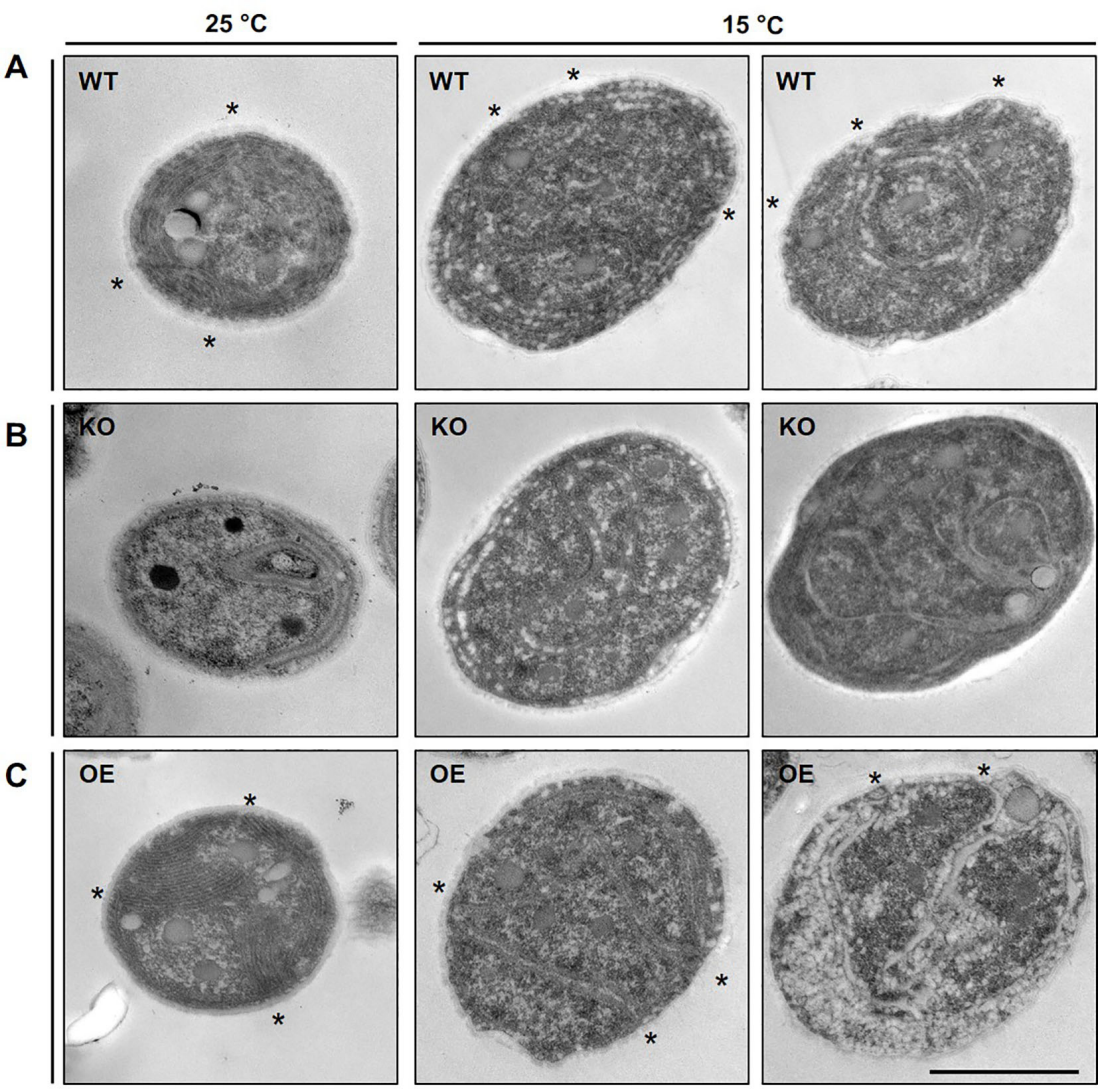
Transmission electron micrographs of Synechocystis cells grown at 25 °C and 15 °C. Representative ultrastructure of *Synechocystis* WT **(A)**, KO **(B)** and OE **(C)**. Black asterisks (*) indicate thylakoid convergence zones. WT, wildtype; OE, *CurT* overexpression; KO, *CurT* knock-out. Scale bar = 1 µm.

Finally, ultrastructural analyses documented that a temperature shift from 25 °C to 15 °C induces a pronounced spatial reorganization of the membrane system in WT and OE cells (**Figure 3**; **Supplementary Figure 2**). While WT cells grown at 25 °C show the typical parietal arrangement of TMs in multiple closely appressed layers adjacent to the PM, as well as TCZs, exposure to cold stress, however, leads to a markedly disorganized thylakoid architecture (**Figure 3A**). Nevertheless, at 15 °C, ordered thylakoid sheets remain detectable near the PM and extend towards it in WT and OE, indicating the formation of TCZs (**Figures 3A, C**). However, TM sheets of cold-treated WT and OE exhibit reduced membrane appression and increased inter-thylakoid spacing, similar to KO cells (**Figures 3A, C**). In addition, cold-treated WT cells (**Figure 3B**) contain perturbed TMs and concentric membrane structures traversing the cytoplasm, resembling the *CurT* KO phenotype under 25 °C control conditions (**Figure 3A**). Meanwhile, the KO line forms typical disordered membrane networks (Heinz et al., 2016; Ostermeier et al., 2026) at both 25 °C and 15 °C (**Figure 3B**), thus displaying the weakest cold-related TM restructuring.

This data strongly suggests that cold-induced reduction of CurT to 58% of the WT level at 25 °C compromises TM organization under low-temperature conditions, consistent with CurT’s proposed role in membrane curvature formation at nascent convergence sites. Strikingly, although the OE line still accumulates higher CurT levels than the WT at 15 °C (**Figures 1A, B**), it also displays severe ultrastructural alterations while accumulating larger amounts of low electron-density cellular compounds in some cases (**Figure 3C**). Finally, at 15 °C, all examined cells showed a significant increase in cell size compared with growth at 25 °C (**Supplementary Figure 1**).

## Discussion

CurT/CURT1 is a key regulator of thylakoid architecture and divisome formation in chloroplasts and cyanobacteria (Armbruster et al., 2013; Heinz et al., 2016; Dann et al., 2025). Our findings demonstrate that cold stress causes severe changes in TCZ marker protein abundance, growth behavior, photosynthetic performance, and importantly TM organization in the cyanobacterium *Synechocystis*.

On the protein level, the TCZ-associated PSII assembly and thylakoid membrane-attachment factors PratA and AncM show a pronounced increase across all CurT expression levels under cold stress (**Figures 1A, B**). This is in line with a previously proposed role of TCZs in PSII assembly/repair (Ostermeier et al., 2025), as PSII repair has been demonstrated to be impaired under cold stress conditions (Kanervo et al., 1997; Allakhverdiev and Murata, 2004), likely necessitating enhanced PSII repair capacities at low temperatures, which appears to be largely independent of CurT abundance.

Interestingly, at 15 °C, OE cells retain D1 levels comparable to WT control levels at 25 °C, while WT D1 levels at 15 °C are clearly diminished (**Figures 1A, B**). This persistence of D1 levels in the OE strain represents one of the few CurT-dependent differences that remains evident under cold stress. Elevated CurT abundance may therefore promote PSII core subunit accumulation even when low temperature imposes strong constraints on protein synthesis and PSII repair. Whether this effect results from enhanced PSII assembly, increased D1 stability, improved repair efficiency, or indirect effects on thylakoid membrane organization remains unclear and warrants further investigation.

Moreover, previous studies extensively tracing transcriptomic and proteomic environmental stress responses in *Synechocystis* have not hitherto pointed towards CurT as a prominently regulated factor under cold-stress. However, such studies have defined cold-stress inducing low temperatures as 20-22 °C (Sinetova and Los, 2016; Mironov et al., 2019), which indicates that CurT function may become critical only under more severe cold stress conditions.

OD_720_-based culture growth measurement points towards a partial convergence of WT and *CurT* KO performance under low temperatures, while subtle growth advantages of OE strains observed under control conditions are diminished at 15 °C (**Figures 1C, D**). This is reflected by OE mutant cultures reaching the highest final OD_720_ of all strains under control conditions, but the lowest maximum OD_720_ at 15 °C (**Figures 1C, D**), pointing towards a physiological trade-off which stabilizes CurT abundance at WT levels despite growth advantages due to elevated expression levels under optimal temperatures.

Culture pigmentation data indicates equilibration of cellular light energy absorption capacities of WT, KO, and OE strains under cold stress (**Figure 2A**). This effect is likely caused by cellular depletion of Chl a and phycobiliproteins in WT and OE as previously described for other cyanobacterial systems exposed to cold stress (Kumar et al., 2011; Mackey et al., 2013), resulting in a convergence towards elevated relative carotenoid contents as previously described for *CurT* KO mutants (Heinz et al., 2016). Cold-induced changes in photosynthetic pigment content are accompanied by reductions in apparent fluorescence-derived yield parameters (**Figure 2B**). Because fluorescence measurements in cyanobacteria are influenced by PSI fluorescence, phycobilisome coupling, and photosystem stoichiometry, these changes cannot be exclusively attributed to altered intrinsic PSII photochemical efficiency. Intriguingly, quantum yield of the OE strain, which was observed to be the highest under control conditions, was found the lowest among all strains throughout at 15 °C (**Figure 2B**), further pointing towards a physiological penalty incurring upon failure to downregulate CurT at low temperatures.

Under control conditions, OJIP chlorophyll fluorescence transients revealed an increased J-step (≈2 ms) in the KO strain compared to WT and OE (**Figure 2C**), which indicates a more reduced PQ pool in KO cells (Tóth et al., 2007) resembling effects observed in tobacco leaves under cold stress (Wei et al., 2022). In addition, in KO mutants, the P peak was delayed, as has previously been observed in WT material under cold stress (Maksimov et al., 2017).Such delay suggests a limitation in downstream electron transport, as the I–P phase reflects the reduction of PSI-side electron acceptors and the progressive saturation of the electron transport chain (Schansker et al., 2005). The observed changes in fluorescence characteristics and electron transport-related parameters already being present in the KO mutant under control conditions point towards a photosynthetic impairment similar to cold-stress symptoms of WT material. The convergence of OJIP transients between WT, OE, and the KO strain under cold stress underlines that CurT is involved in safeguarding PSII activity and maintaining electron transport efficiency under optimal growth conditions, while its functional contribution appears diminished under low-temperature conditions.

Besides TCZ-related protein accumulation, growth, and photosynthetic performance, the cold-stress regime applied caused pronounced ultrastructural rearrangement of the studied *Synechocystis* cell material. Under cold stress and upon corresponding depletion of cellular CurT levels (**Figures 1A, B**), WT TMs were found spatially redistributed, with increased membrane spacing and protrusion of loop-like individual thylakoid sheets towards the cell interior resembling *CurT* KO mutant thylakoid distribution (**Figure 3**). Meanwhile, the OE strain retained a more angulated and appressed thylakoid architecture even at 15 °C, more closely resembling the control WT.

Under cold stress, thylakoid membranes are spatially redistributed within the cell, with reduced stacking at the cell periphery, increased inter-thylakoid spacing, and the formation of concentric or tubular membrane structures inside the cytosol. These cold-induced reorganizations may reduce membrane coupling and thus excitation pressure exerted on the photosynthetic electron transport chain in WT, representing an adaptive response to temperature-limited electron sink capacity, which in turn could ensure protection of the photoinhibition-sensitive PSII.

The partial phenotypic convergence of WT, KO, and OE lines at 15 °C suggests that low temperature imposes a common physiological bottleneck that attenuates many CurT-dependent differences. Notably, however, D1 accumulation in the OE strain remains elevated relative to WT under cold stress, indicating that specific CurT-dependent effects persist despite the overall convergence of physiological and ultrastructural phenotypes. At the same time, the strong similarity between cold-treated WT cells and the CurT-deficient phenotype supports a model in which CurT contributes to membrane robustness when thylakoid fluidity and curvature are challenged.

Further, ultrastructural analysis on WT indicates that, even though cellular CurT is depleted and membrane architecture is dynamically reorganized in *Synechocystis* upon cold stress, TCZs formation persists (**Figures 3A, C**). This is also shown by an enhanced level of the membrane anchor AncM under 15 °C (Ostermeier et al., 2022)(**Figures 1A, B**), which points towards a functional role of TCZs that remains relevant also under cold stress and suggesting that core CurT-dependent structures are maintained upon lowering nearly halve of cellular CurT levels. The latter is in line with observations in functional *CurT* knock-down strains showing serious depletion in cellular CurT while still forming TCZs (Ostermeier et al., 2026).

In summary, the presented data suggests that upon cold stress, many CurT-dependent physiological and ultrastructural differences become attenuated. Nevertheless, the persistence of elevated D1 accumulation in the OE strain indicates that CurT retains a measurable influence on PSII-related processes even under conditions where most other strain-specific phenotypes converge. Both photosynthetic deficiencies and maximum culture density at OD_720_ appear near WT-like, while cell duplication time remains compromised. The latter is in line with a separate role of CurT in cell division besides TCZ formation and adjacent processes as recently proposed (Dann et al., 2025; Zhang et al., 2025).

## Data Availability

The datasets presented in this article are not readily available because there are no restrictions. Requests to access the datasets should be directed to m.ostermeier@bio.lmu.de.
