# Supplementary material for: Cold stress impairs CurT accumulation and thylakoid architecture in Synechocystis sp. PCC 6803
Source: Front Plant Sci. 2026 Jun 26;17:1876046. doi: 10.3389/fpls.2026.1876046 (PMC13353191; doi:10.3389/fpls.2026.1876046)
Supplement: Supplementary file 1 [file DataSheet1.pdf]

Supplemental Figure 1

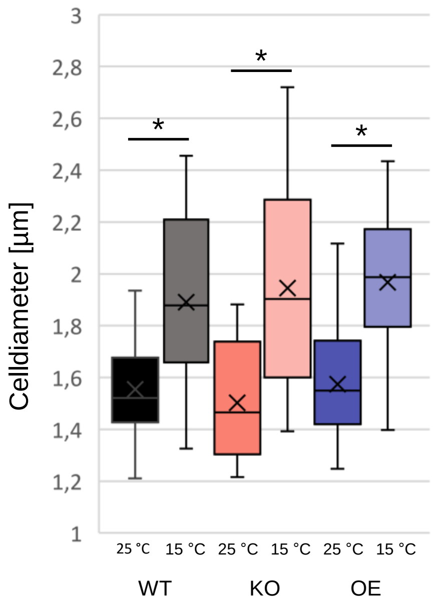

Supplmental Figure 1: Alterations in cell diameter. For every 10 to 15 cells, 2 measurement points were recorded. Significant differences according to Student’s t test (two-sided, $p<0.01$) are indicated by asterisks.

Supplemental Figure 2

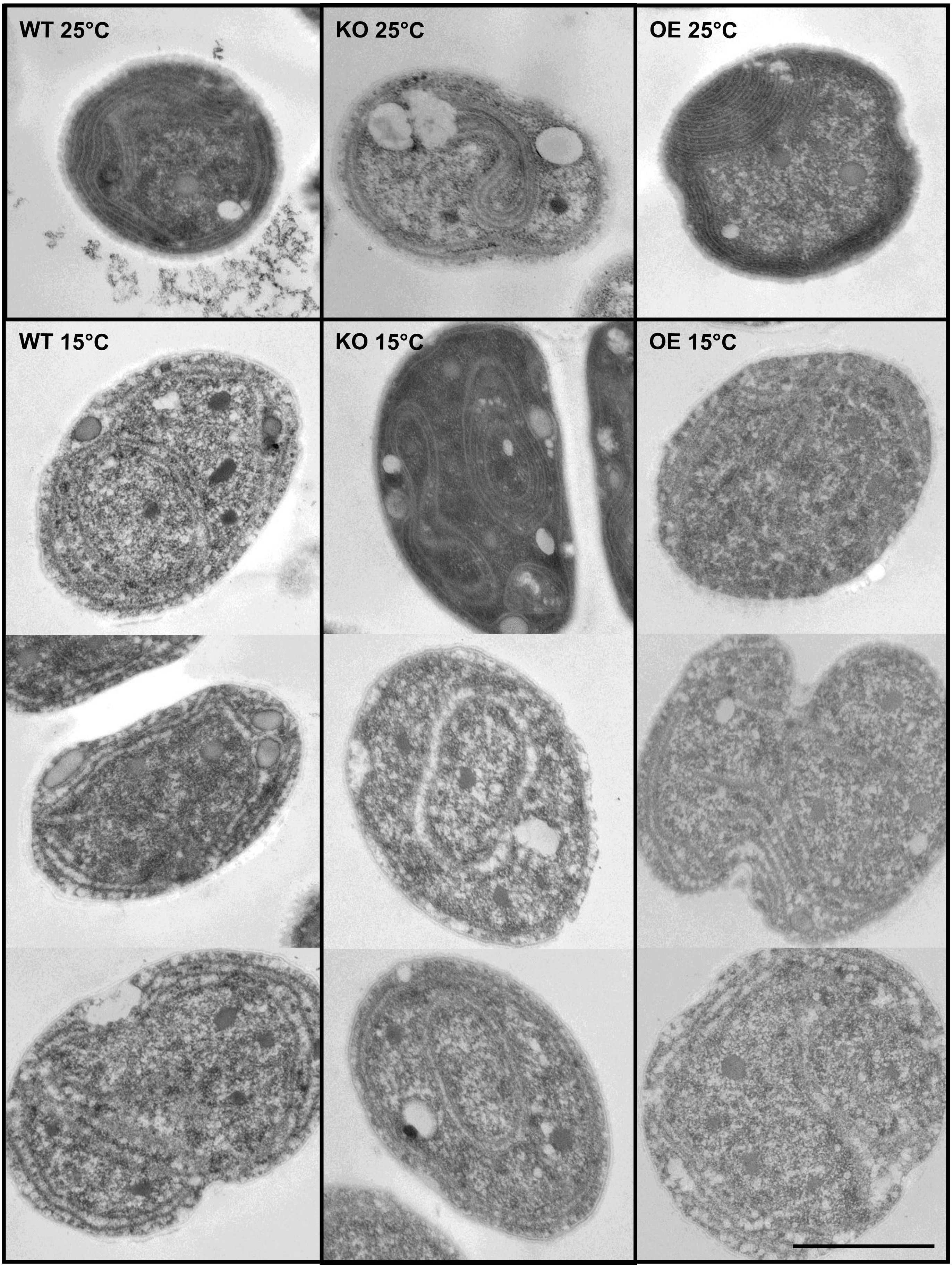

Supplemental Figure 2: Additional transmission electron micrographs of Synechocystis WT, KO and OE. Bar = 1 µm.
